# A novel strategy for difficult biliary cannulation using cold resection of the oral protrusion before needle-knife precutting

**DOI:** 10.1055/a-2944-3526

**Published:** 2026-09-04

**Authors:** Kotaro Takeshita, Kensei Kawasaki, Aoi Koizumi, Kaho Takagi, Takuya Okamoto, Kenji Matsuo, Satoshi Asai

**Affiliations:** 1 Department of Gastroenterology 38434 Tane General HospitalOsakaJapan

When wire-guided cannulation (WGC) and pancreatic guidewire-assisted cannulation (PGW) fail, alternative biliary access techniques are needed. During needle-knife precutting (NKP), inadequate separation of the incised mucosa may obscure the biliary orifice. We describe the use of cold resection of the oral protrusion (CROP) before NKP (CROP-NKP) as a rescue technique for difficult biliary cannulation after failed conventional approaches. 1​ ​2​

**Fig. 1:**
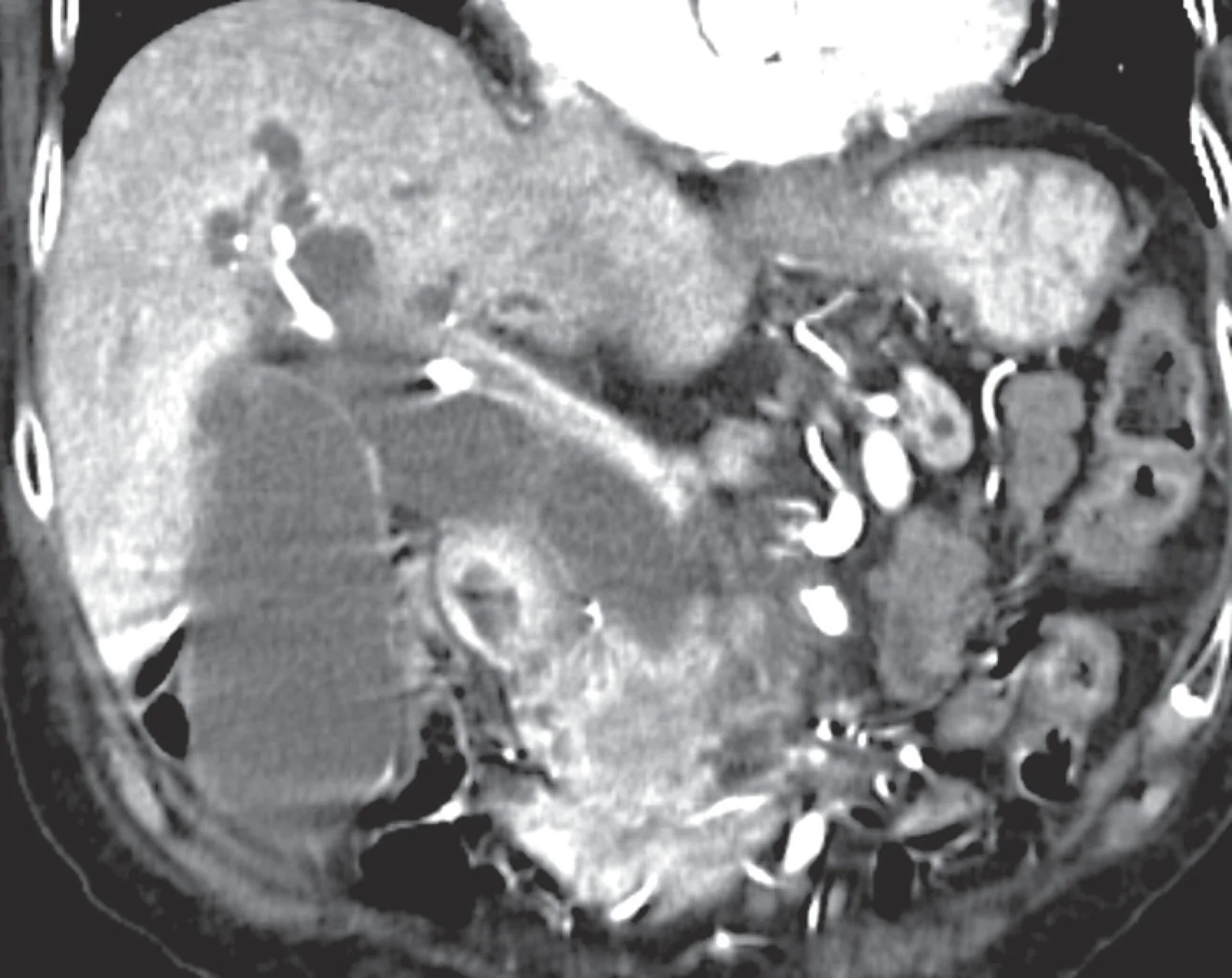
Contrast-enhanced computed tomography shows distal biliary obstruction due to pancreatic cancer. The proximal bile duct is dilated.

**Fig. 2:**
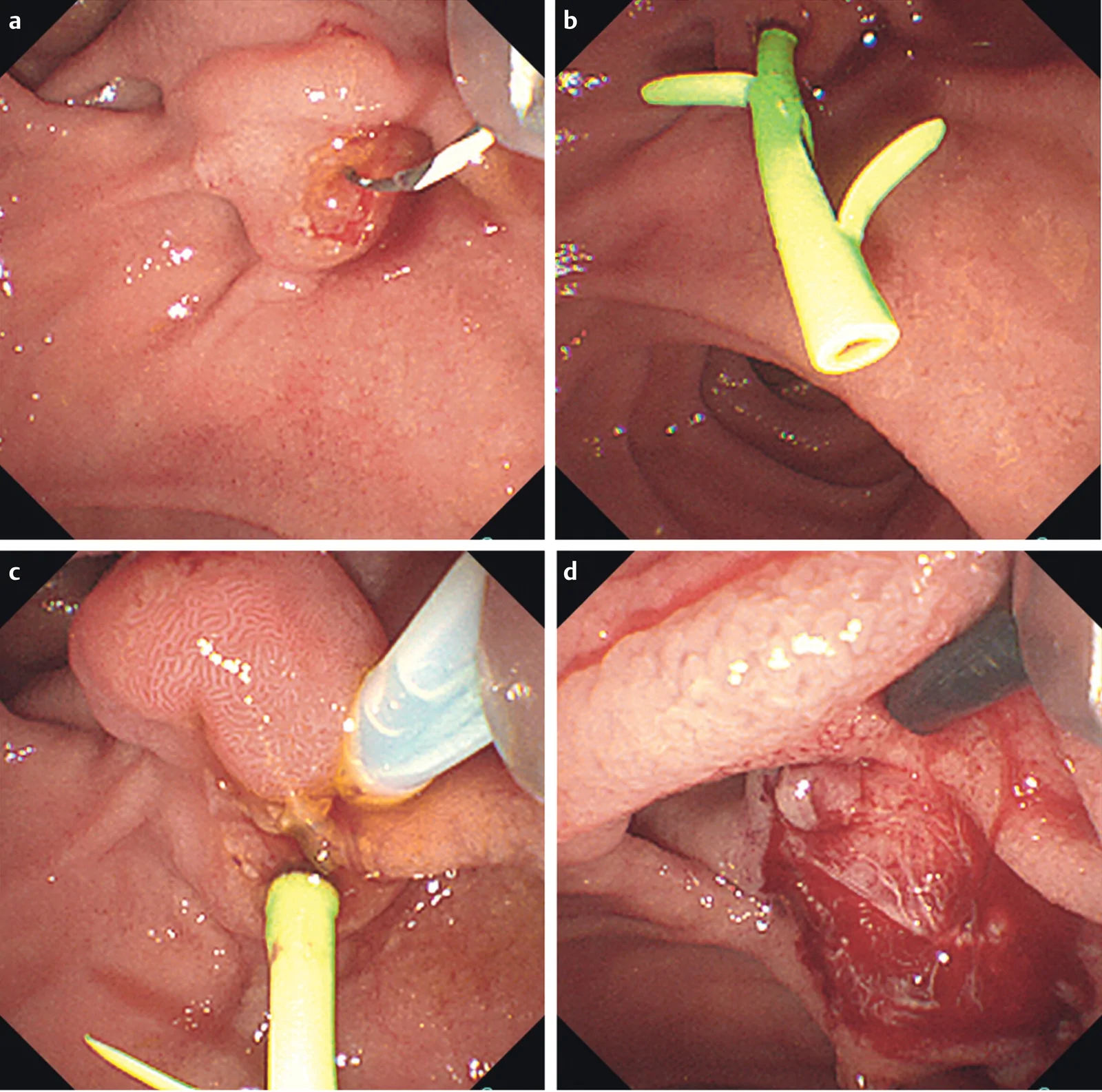
( **a** ) Failure of wire-guided cannulation and pancreatic guidewire-assisted cannulation. ( **b** ) A pancreatic plastic stent is placed. ( **c** ) The duodenal mucosa overlying the oral protrusion is captured with a 15-mm braided snare (SnareMaster; Olympus, Tokyo, Japan). Conventional cold-snare closure failed to transect the mucosa. ( **d** ) Mechanical transaction is completed without electrocautery by drawing the snared tissue into the working channel. Mild oozing is observed and controlled with irrigation alone.

An 86-year-old woman with a performance status of three presented with obstructive jaundice due to pancreatic head cancer ( Fig. 1 ). Given her frailty, biliary drainage with a fully covered self-expandable metal stent (FCSEMS) was planned. WGC and PGW failed ( Fig. 2a , Video 1 ). A duodenal diverticulum, overlying folds, and redundant mucosa made NKP difficult.

**Video 1** Successful biliary cannulation was achieved using the cold resection of the oral protrusion followed by a needle-knife precutting technique after failed conventional cannulation including wire-guided cannulation and pancreatic guidewire-assisted cannulation.


After pancreatic stent placement ( Fig. 2b ), resection was confined to the duodenal mucosa overlying the oral protrusion. Duodenal mucosa beyond its margins lacked support from the oral protrusion and was not captured because resection was considered unsafe. The mucosa was captured with a 15-mm braided snare (SnareMaster; Olympus, Tokyo, Japan; Fig. 2c ).

**Fig. 3:**
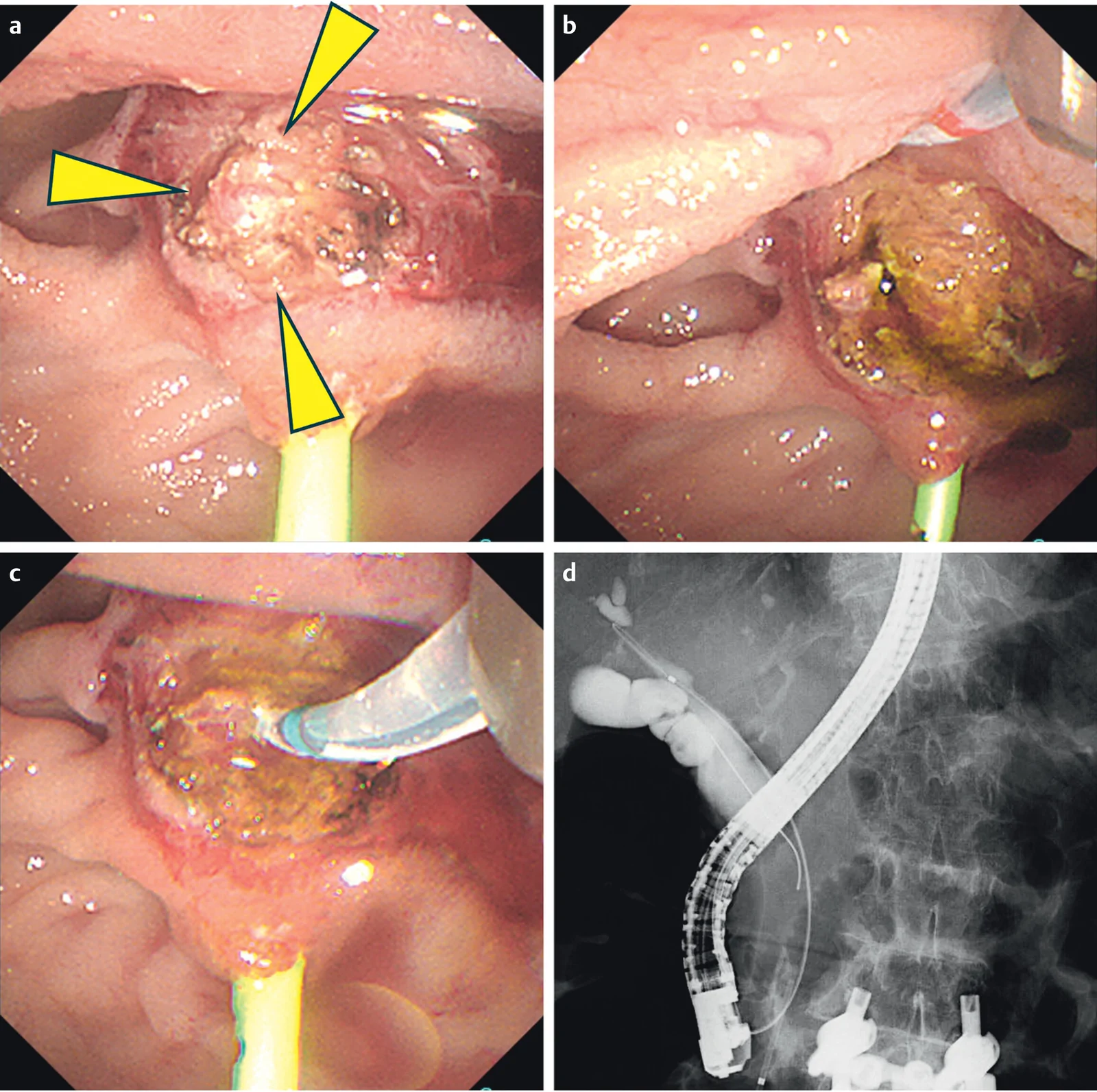
( **a** ) After irrigation, the bulging area suggestive of the sphincter of Oddi is identified within the exposed ulcer base. ( **b** ) The bulging area is incised with a needle-knife, and bile outflow is established. ( **c** ) Catheter insertion through the incision achieves deep biliary cannulation. ( **d** ) Fluoroscopy shows severe distal biliary stenosis.

Conventional cold-snare closure failed to transect the mucosa; the snared tissue was therefore drawn into the working channel, thereby completing mechanical transection without electrocautery ( Video 1 ). Had this failed, we would have repositioned the snare and recaptured the tissue without electrocautery. Mild oozing resolved with irrigation ( Fig. 2d ). A bulge suggestive of the sphincter of Oddi was identified and incised ( Fig. 3a, b , Video 1 ).

**Fig. 4:**
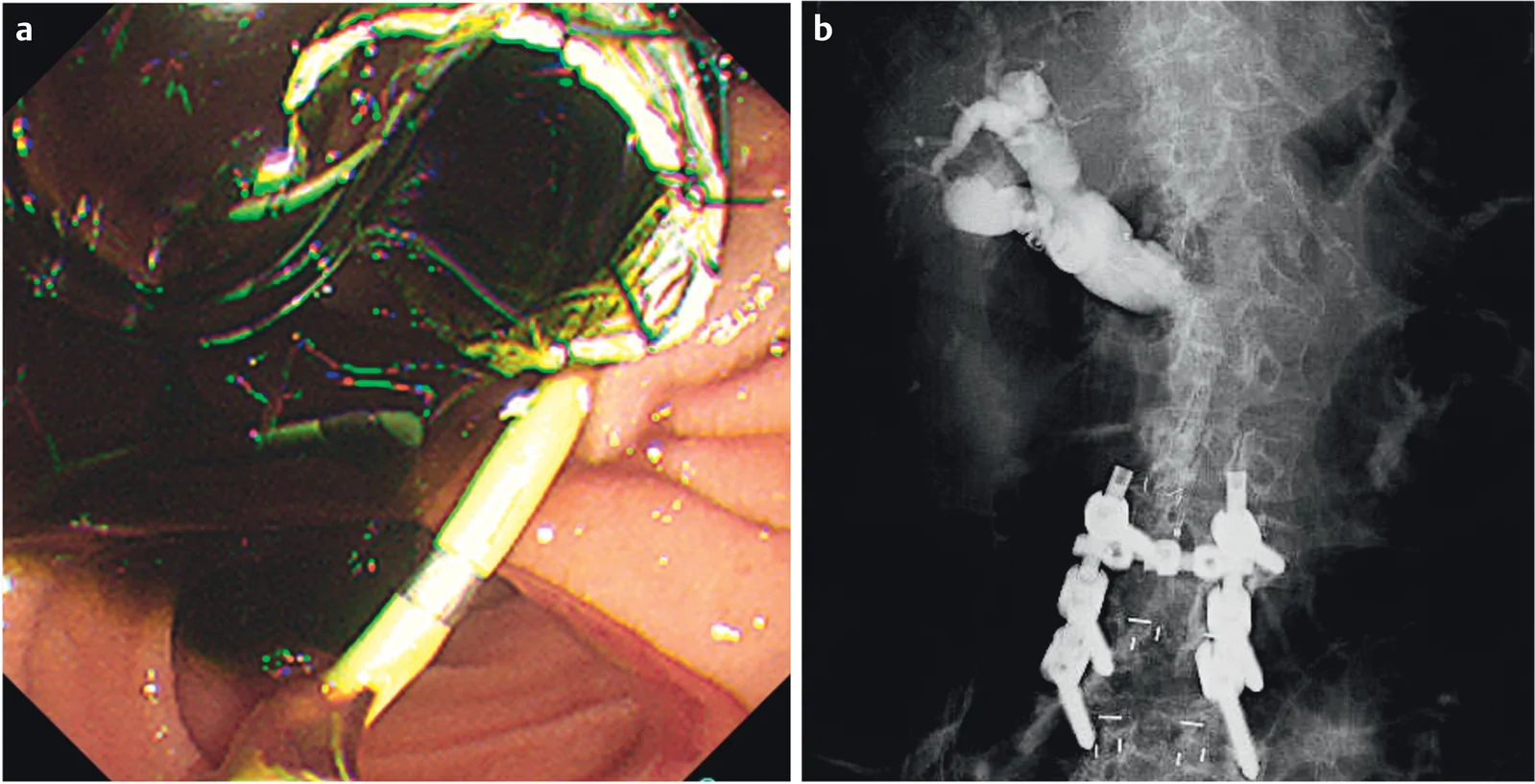
( **a** ) A fully covered self-expandable metal stent (10 mm×60 mm, HANARO stent; M.I. Tech, Seoul, Korea) is deployed. ( **b** ) A fully covered self-expandable metal stent is placed.

Deep biliary cannulation was achieved within 8 minutes ( Fig. 3c, d ). Cholangiography showed distal stenosis ( Fig. 4a ), and an FCSEMS (10 mm×60 mm, HANARO stent; M.I. Tech, Seoul, Korea) was deployed ( Fig. 4 ). No adverse events occurred.

Although partial ampullary endoscopic mucosal resection has been reported, available evidence remains limited. 1​ ​2​ By limiting resection to the duodenal mucosa overlying the oral protrusion and avoiding electrocautery, CROP-NKP may improve exposure while potentially reducing thermal injury; however, ampullary safety remains unproven 3​ 4​ ​5​ .
